# What is missing in the study of emotion expression?

**DOI:** 10.3389/fpsyg.2023.1158136

**Published:** 2023-04-27

**Authors:** Elisa Straulino, Cristina Scarpazza, Luisa Sartori

**Affiliations:** ^1^Department of General Psychology, University of Padova, Padova, Italy; ^2^IRCCS San Camillo Hospital, Venice, Italy; ^3^Padova Neuroscience Center, University of Padova, Padova, Italy

**Keywords:** expressed emotion, kinematics, face perception, laterality of motor control, happiness, fear, anger, dynamic patterns

## Abstract

While approaching celebrations for the 150 years of “The Expression of the Emotions in Man and Animals”, scientists’ conclusions on emotion expression are still debated. Emotion expression has been traditionally anchored to prototypical and mutually exclusive facial expressions (e.g., anger, disgust, fear, happiness, sadness, and surprise). However, people express emotions in nuanced patterns and – crucially – not everything is in the face. In recent decades considerable work has critiqued this classical view, calling for a more fluid and flexible approach that considers how humans dynamically perform genuine expressions with their bodies in context. A growing body of evidence suggests that each emotional display is a complex, multi-component, motoric event. The human face is never static, but continuously acts and reacts to internal and environmental stimuli, with the coordinated action of muscles throughout the body. Moreover, two anatomically and functionally different neural pathways sub-serve voluntary and involuntary expressions. An interesting implication is that we have distinct and independent pathways for genuine and posed facial expressions, and different combinations may occur across the vertical facial axis. Investigating the time course of these facial blends, which can be controlled consciously only in part, is recently providing a useful operational test for comparing the different predictions of various models on the lateralization of emotions. This concise review will identify shortcomings and new challenges regarding the study of emotion expressions at face, body, and contextual levels, eventually resulting in a theoretical and methodological shift in the study of emotions. We contend that the most feasible solution to address the complex world of emotion expression is defining a completely new and more complete approach to emotional investigation. This approach can potentially lead us to the roots of emotional display, and to the individual mechanisms underlying their expression (i.e., individual emotional signatures).

## Introduction

*“…Facial movement of expression impresses us through its changes, through its melody.*

*The characteristic of the person will always be the way they move, the melody of the expression; this can never be caught in snapshots…”*

(Sir Ernst Gombrich, cited by Miller, 1983)

According to a growing crowd of influential researchers, we are now on the edge of a radical change of perspective about how we think of emotions (Adolphs et al., 2019; Barrett et al., 2019; Heaven, 2020). Emotion is one of the building blocks of human life. However, how humans dynamically and genuinely express their emotions with the whole body is still little investigated.

Over the last century, the science of emotion gradually anchored to six prototypical facial expressions: anger, disgust, fear, happiness, sadness, and surprise (i.e., the basic six; Ekman et al., 1969; Ekman and Friesen, 1971). These six expressions were described as unitary entities and conceived as if they were mutually exclusive categories. In particular, Basic emotion theory proposes that a limited number of emotions are manifested through organized and recurrent patterns of behavior in a kind of “one-to-one” correspondence, conserved by evolution to handle basic life situations (Ekman, 1992a,b, 2003; LeDoux, 1995, 2012; Ekman and Cordaro, 2011; Damasio and Carvalho, 2013). Jack et al. (2016) further narrowed this view by suggesting a four-scheme model of expression, each of which communicates a specific combination of valence, arousal and dominance, probably evolved from a simpler communication system (see also the “Three Primary Color Model of Basic Emotions,” Gu et al., 2019). Needless to say, all these elegant models leave most human expressions unexplored (Barrett, 2017; Adolphs et al., 2019; Barrett et al., 2019). Moreover, the term basic seems to underlie that the emotions are discrete, rather than a family of related states (Scherer and Ellgring, 2007; Tracy and Matsumoto, 2008; Roseman, 2011; Bänziger et al., 2012; Keltner and Cordaro, 2017; Cordaro et al., 2018). Instead of considering happiness as a single emotion, for instance, research should try to unpack emotional categories into their components: the happiness umbrella might cover joy, pleasure, compassion, pride, and so on. According to the Constructionist theory, a wide range of emotions have evolved, shaped by language and cognitive appraisal (Russell and Barrett, 1999). All the emotions can be located in a circle called circumplex (Russell, 1980), characterized by different amounts of valence (pleasure/displeasure axis) and arousal (high/low axis). Basic emotion and Constructionist theories have been pitted against each other for more than a century in the so-called 100-year war (Lindquist et al., 2013; for a review see Crivelli and Fridlund, 2019). Now, thanks to modern neuroscience, we are finally beginning to understand the complexity of the emotional world. Emotional expression might be far richer and more complex than the prototypical patterns of facial muscle movements so far considered.

The article is not intended to be a comprehensive review on emotions, but rather a focused review on the expression of emotions by presenting critical research suggesting that humans dynamically perform genuine and mixed expressions with face and body. We also propose a new integrated model capable of extracting multimodal algorithms applicable in ecological contexts for the assessment of inter-individual and cultural differences.

## Distinct and independent pathways for posed and genuine facial expressions

Humans have 43 facial muscles, with which they can produce up to 10,000 different expressions, making the human face one of the most powerful communicative tools our species has (Rinn, 1984). Note that even the expression of the same emotion may be conveyed by different neural systems. For instance, two anatomically and functionally different neural pathways sub-serve the expressions of genuine and posed facial expressions. The contraction of mimic muscles related to genuine emotion originates from subcortical brain areas that provide excitatory stimuli to the facial nerve nucleus in the brainstem via extrapyramidal motor tracts, which often involve the concomitant contraction of the ocular orbicular muscles. In contrast, posed smiles are controlled by impulses of the pyramidal tracts from the motor cortex (Frank et al., 1993; Schmidt et al., 2006; Sidequersky et al., 2014). Therefore, we have different pathways for posed (i.e., voluntarily controlled) and spontaneous (i.e., involuntarily produced) facial displays (Ross et al., 2016), so that the genuine pathway has been associated with more synchronized, smooth, and symmetrical expressions compared to the pyramidal voluntary system (Ross et al., 2019). Posed facial expressions are those displayed intentionally by a person who pretends to transmit a specific emotion (Namba et al., 2017), while spontaneous facial expressions are those elicited by true emotional content and usually correspond to a more genuine emotional experience (Niedenthal et al., 2010; Zloteanu and Krumhuber, 2021). For example, a smile is genuine when listening to a joke. However, people also try to smile when they feel angry, scared, tired or embarrassed, to hide these emotions in contexts where they are inappropriate. Notably, the upper face muscles (i.e., eye areas) are mainly controlled by the subcortical and extrapyramidal systems, whereas the lower face (i.e., mouth area) is under the voluntary control of the motor system (Gazzaniga and Smylie, 1990; Hopf et al., 1992; Ross et al., 2007; Krippl et al., 2015). This means that: (i) facial blends of expressions might occur across the horizontal axis (i.e., eyes vs. mouth areas, Ross et al., 2016); and (ii) muscles of the upper face are innervated bilaterally, whereas muscles of the lower face are cross-innervated prevalently from the contralateral side (Morecraft et al., 2004; Ross et al., 2016). Therefore, small changes in the dynamical development of a facial display may characterize and distinguish genuine from posed facial expressions, a topic still poorly investigated (but see Sowden et al., 2021). Investigating the relative contributes of upper and lower facial cues to emotion recognition is also particularly interesting in the light of the current COVID-19 pandemic. By 2020, medical facemasks occluding the lower portion of the face have become a pervasive feature in everyday life. These masks are clearly designed for preventing infection. However, there are concerns related to their possible impact on emotion recognition. Results from a just-published study (Marini et al., 2021) show that mask-wearing has two problematic side effects. First, by making the mouth invisible, they interfere with the recognition of emotional states. Moreover, they compromise facial mimicry reducing therefore emotional contagion (Dimberg et al., 2011; Hess and Fischer, 2013; Tramacere and Ferrari, 2016; Palagi et al., 2020).

When a genuine emotion is experienced, the expression of this emotion cannot be totally inhibited or modified, and it follows a rather stereotyped pattern (Baker et al., 2016). For example, a genuine smile – like automatic movements - can appear as fast as 0.30 s, and it usually fades away after 3 to 4 s (Schmidt et al., 2003). However, the diversity of appearance and dynamics of spontaneous smiles still requires a better understanding of a smile’s properties and patterns, to determine what features or temporal parameters are key in transmitting information and how they variate in different contexts (Schmidt et al., 2003). What is needed to make sense of emotional expressions is therefore a much richer taxonomy.

## The missing piece: genuine and dynamic displays

Experimental studies of emotional expressions, inaugurated by Ekman (1965) and continued by a large number of scholars, have focused on static and prototypical facial expressions with the goal of finding evidence for a universal theory of emotional expressions (with the help, for example, of facial expression analysis or FACS; Ekman et al., 2002). Past research, moreover, has favored the use of stereotyped emotional stimuli over more ecologically valid but less controllable expressions in order to better investigate the influence of variables such as gender, age, and personality traits. It was possible to show, for example, that observed emotion recognition performance is higher in females (e.g., Hoffmann et al., 2010; Saylik et al., 2018; Wingenbach et al., 2018; Connolly et al., 2019), decreases with age (e.g., Ruffman et al., 2008; West et al., 2012; Abbruzzese et al., 2019), and is particularly affected in people with alexithymia (e.g., Vermeulen et al., 2006; Scarpazza et al., 2014; Starita et al., 2018; Malykhin et al., 2023). The use of static, posed, and archetypical facial expressions has provided in fact high scientific control and repeatability, but at the cost of data variability that likely accounts for actual emotional manifestations (Kret and De Gelder, 2012). Past researchers have tried to balance these research priorities and have correctly identified the optimal ways of expressing different emotions (e.g., Ekman and Friesen, 1976; Wallbott and Scherer, 1986; Motley and Camden, 1988; Russell, 1994; Biehl et al., 1997; Tcherkassof et al., 2013). Now we can try to get on their shoulders and break down the question further, to investigate the distinction between authentic and posed emotions. It is time to increase complexity. We certainly do not deny the fact that using genuine and ecologically valid emotional stimuli will still have some problems too. For instance, showing combinations of different emotions might add complexity to the task of identifying emotional expressions. Indeed, as the prototypical quality of the stimuli lessens, the influence of perceiver-based processes (e.g., her/his current emotional state) becomes more prominent in the perceptual judgement process. We might ask: At what point does the benefit of using ecologically valid stimuli balance out the inverse increase in the influence of perceptual-based factors? We are aware that emotion science is now facing a classic trade-off between external validity of stimuli and their recognition properties. We believe, however, that if the science of emotions were to remain still anchored in prototypical displays, it would not rise to the level of understanding the recognition processes evolved in response to real stimuli during the phylogenetic development of the human species. Having a comprehensive taxonomy of real emotion expression will help to formulate new theories with a greater degree of complexity. Recently, some dataset including spontaneous emotions “in the wild” have been released (Guerdelli et al., 2022). However, the majority of emotional facial data sets of stimuli used in scientific research are based on static photographs of non-spontaneous facial expressions (Tcherkassof et al., 2013; O’Reilly et al., 2016; Dawel et al., 2022). This methodology has been questioned given the low generalizability of its results (Russell, 1994; Tcherkassof et al., 2013). People project their stereotypes in posed expressions, their common view of what they believe an emotional facial expression should look like (e.g., a scowling facial configuration to express anger), but these displays do not necessarily correspond to how people actually behave in real life (Barrett et al., 2019).

Genuine expressions differ specifically from posed expressions in both temporal and morphological features (Wehrle et al., 2000; Cohn and Schmidt, 2004; Sato and Yoshikawa, 2004; Ekman and Rosenberg, 2005; Yoshikawa and Sato, 2006; Valstar and Pantic, 2010). In first instance, genuine facial expressions can occur within a fraction of a second (i.e., micro expressions; Ekman, 2009). In second instance, they are usually less intense and more subtle than posed expressions classically used in laboratory (Tcherkassof et al., 2013). This disparity could explain why the recognition accuracy of posed emotions, characterized by prototypical and very intense facial configurations, is much higher than that of spontaneous emotions (Barrett et al., 2019). Thus, more genuine stimuli are needed in research. Unfortunately, such databases are still rare because of the practical (e.g., the methodology needed to collect these stimuli) and ethical difficulties (see Philippot, 1993 for initial considerations) of documenting and collecting genuine expressions (Tcherkassof et al., 2013). In fact, it is difficult to trigger authentic emotions with the same intensity as fake emotions and to validate the resulting dataset of posed and spontaneous displays (Krumhuber et al., 2017). For example, some datasets built on the performance of professional actors did not verify whether the expressions were then perceived as genuine by observers (McLellan et al., 2010). And even when it was verified, it missed the next step, which was to cross-reference the observers’ scoring with the emotion actually experienced by the actor (Dawel et al., 2017). Only recently, a dataset of authentic and inauthentic emotional expressions matched the emotion felt by the actor with that perceived by the observer in terms of intensity and genuineness (Miolla et al., 2022). The next step will be to create dataset including also the context of the emotional display. In fact, posed expressions often occur in everyday life (e.g., when mothers exaggerate their facial movements to be perceived accurately by their infant children) and they are nonetheless genuine and appropriate to the context.

A related problem in the study of emotions expressions is that the majority of the literature have employed static facial stimuli (Tcherkassof et al., 2007; McLellan et al., 2010, 2012; Douglas et al., 2012; Li et al., 2012; Dawel et al., 2015). Only the peak intensity of emotions was usually shown, while the time-course of facial expressions was substantially ignored. However, facial expressions are not an all-or-nothing phenomenon: the nature of facial expressions is that they are dynamic in presentation (Rymarczyk et al., 2019). Recent literature suggests that dynamic displays enhance the ability not only to correctly recognize facial expressions (Cunningham and Wallraven, 2009; Ceccarini and Caudek, 2013; Krumhuber et al., 2013), but also to discriminate genuine and posed facial expressions of emotion (Krumhuber et al., 2013; Namba et al., 2018, 2021; Lander and Butcher, 2020) and to elicit stronger muscle activation during mimicry (Rymarczyk et al., 2016). The use of dynamic emotional stimuli is more ecologically valid (Bernstein and Yovel, 2015), as an emotional message is usually reflected in dynamic complex action patterns and not in static facial clues (Tcherkassof et al., 2013; O’Reilly et al., 2016). This is probably because dynamic faces can transmit an evolving hierarchy of signals over time (Delis et al., 2016), thus providing much more information than static pictures (e.g., time course, change of speed, facial-feature amplitude, and irregularity of an expression; Tcherkassof et al., 2013). This effect has also been confirmed by the activation of a broader neural network in the observer when using dynamic stimuli compared to static emotion stimuli (Ambadar et al., 2005; Weyers et al., 2006; Trautmann et al., 2009). Only recently, an increasing number of dynamic emotion data sets have been developed, including, for instance, the Cohn–Kanade AU-Coded Facial Expression Database (Kanade et al., 2000; Lucey et al., 2010) and the Video Database of Moving Faces & People (O’Toole et al., 2005; for a review see Krumhuber et al., 2017). However, an aspect that has been largely neglected is the key role of temporal dynamics as a locus for investigating the encoding of facial displays. To date, little is known about the temporal course of facial expressions (Tcherkassof et al., 2013). Temporal parameters, such as the apex period (i.e., the time duration before the peak intensity starts decreasing) and movement time (i.e., the time from facial display onset until it disappears) of facial expressions, might allow unveiling the secret syntax of emotional language. For instance, recent research has shown that eyelid movements precede eyebrow movements in genuine surprise displays (Namba et al., 2017) and this could help to differentiate spontaneous from simulated expressions. In the case of smiles, shorter durations and more irregular onset have been associated with lower perceived genuineness (Krumhuber et al., 2013).

To sum up, research on emotion expression has been extensively conducted during passive observation of posed and static pictures (e.g., Karolinska Directed Emotional Faces; Lundqvist et al., 1998). More ecological and dynamic stimuli such as spontaneous recordings from real-time interactions have rarely been adopted. Crucially, posed expressions have lower ecological validity and differ in timing from spontaneous ones (Ekman and Rosenberg, 2005). Approaches based on static and simulated portrayals may, therefore, fail to generalize to real-world behavior (Zeng et al., 2009). Even distinguishing facial expressions into genuine or posed, depending on the manner and context in which they are produced, may be too simplistic, because they are just the poles of a broad spectrum with various gradations of color.

## Facial blends of emotion: the hemispheric lateralization puzzle

Many — even most — experiences of emotion are complex blends of emotion (Parr et al., 2005; Du et al., 2014). Multiple emotions can occur in a rapid sequence, again and again, or can merge in a mosaic. Humans have the capacity to produce facial blends of emotions in which the upper and lower face simultaneously display different expressions, suggesting that their underlying emotions are compound entities (Larsen et al., 2001; Scherer, 2009). Facial expressions are organized predominantly across the horizontal facial axis (i.e., upper-lower areas), but there are exemplars (e.g., surprise-frown or smile-grimace) in which the expression on the right and left sides of the face differs, thus providing evidence that facial blends of emotions may also occur across the vertical facial axis (i.e., left–right areas). In this vein, three major models of emotional processing address the so-called “hemispheric lateralization of emotions” topic in humans (Demaree et al., 2005; Killgore and Yurgelun-Todd, 2007). The Right Hemisphere Hypothesis asserts that all emotions and their associated expressions are a dominant and lateralized function of the right hemisphere. The Valence Hypothesis states that negative, avoidance or withdrawal-type emotions and their associated expressions are lateralized to the right hemisphere, whereas positive approach-type emotions and their associated expressions are lateralized to the left hemisphere. Finally, the Emotion-type Hypothesis (Ross et al., 2007, 2016) affirms that primary emotional responses are initiated by the right hemisphere on the left side of the face, whereas social emotional responses are initiated by the left hemisphere on the right side of the face. The most striking examples are expressions that display a “double peak” phenomenon (e.g., grimace-smile characterized by an initial movement followed by a slight relaxation and then a second movement to the final peak) as a result of dual or competing hemispheric motor control (Ross et al., 2016). In some instances, the initial movement starts on one side of the face and the second movement starts on the opposite side of the face. For instance, Duchenne and non-Duchenne are terms used to classify if a smile reflects a true emotional feeling versus a false smile (Ekman and Friesen, 1982; Ekman et al., 1988). A felt (Duchenne) smile is very expressive and it is classically described as causing the cheeks to lift, the eyes to narrow and wrinkling of the skin to produce crow’s feet. A false (non-Duchenne) smile, instead, would only involve the lower face area. However, recent research has shown that the difference between a felt (Duchenne) versus a fake smile might in fact be revealed by the side of the face initiating the smile (Ross et al., 2016).

Despite the importance of emotion in human functioning, scientists have been unable to reach a consensus on the debated issue concerning the lateralization of emotions. We believe that investigating the time course of facial blends of emotions, which can be controlled consciously only in part, would provide a useful operational test for comparing the different predictions of various models, thus allowing this long-standing conundrum to be solved.

## Ecological validity needs context

Emotions can be described as responses to events that are important to an individual (Izard, 2010; Mulligan and Scherer, 2012; Ekman, 2016; for an overview see Scherer, 2009). They are usually expressed with the aim to be recognized by the addressee and might be expressed differently depending on who is the interlocutor. In this light, facial expressions are regarded as affective signals, which can convey social information regarding the expresser’s experience of an emotional event (Ekman, 2004; Scherer and Moors, 2019).

One of the hallmarks of social psychology is that in real life situations, body kinematics, gaze-related information, and contextual cues are all critical cues in guiding motor behavior (Sartori et al., 2011; Reader and Holmes, 2016). In the emotional world, the facial display is necessary but may be not sufficient to express and interpret correctly other’s emotions (Barrett et al., 2011). Humans do not interact with ‘bodyless’ or ‘contextless’ faces, as occurs in most of the current research: they constantly receive and integrate multimodal information. Needless to say, a facial expression could be misinterpreted when analyzed independently from the context in which is presented: for instance, tears of victory mean happiness.

Future studies should consider to adopt real-time naturalistic settings: for instance, involving participants in a dyadic interaction (i.e., authentic emotion induction; Zhang et al., 2014), while recording both their movements. Only the adoption of an ecological behavioral approach will allow to genuinely evaluate the effect of social context on emotional functioning. Participants’ spontaneous expressions should be video recorded at high frame rates using specialized recording equipment to provide a good resolution database, allowing the investigation of micro-expressions and subtle temporal features to be matched with self-reported feelings. Introspective measures constitute in fact an essential validation approach, as they provide insight into the elicitation effectiveness (Gray and Watson, 2007). Notably, the study of the neural underpinning of real-time contagious phenomena (e.g., the social transfer of pain) is now extremely relevant, as recently confirmed by Smith and colleagues (Smith et al., 2021). In this perspective, the existing literature has few or null ecologic validity. According to the influential article by Heaven (2020), researchers need to do what Darwin (1859) did for The Origin of Species: “Observe, observe, observe.” Watch what people actually do with their faces and their bodies in real-life contexts. More data and analytical techniques could help researchers to learn something new, instead of revisiting old data sets and experiments (Heaven, 2020).

## Holistic coding: let us take the whole picture

When we are in the grip of an emotion, a cascade of changes occurs in the face, gaze, autonomic nervous system activity, and in our expressive body behavior (for review, see Keltner et al., 2016, 2019). In 2019, Barrett and colleagues published a benchmark review on emotion expression (Barrett et al., 2019). They considered over 1,000 papers and they reached an unambiguous conclusion: the face is not the whole picture. Other aspects, including body movement, gaze, and physiological changes (e.g., cardiovascular changes) are crucial in our expression and perception of emotions. Therefore, a pressing need in the study of emotional expression appears to be necessary to move beyond the narrow focus on facial displays.

In the real world, bodies and faces are almost never perceived in isolation, but rather as an integrated whole. Bodies contain valuable information about the actions and intentions of others, which often intensifies or conversely cancels out the emotion expressed by the face (de Gelder, 2009; Aviezer et al., 2012; de Gelder et al., 2015). When emotion facial expressions are paired with incongruent bodily expressions (e.g., anger facial expression with a fearful body pose), for instance, perceivers show distinct neural responses and impaired recognition, even when they are consciously focusing on the face alone (Meeren et al., 2005; Borgomaneri et al., 2020). These findings suggest that it might be fruitful to focus more attention on the body when considering emotion expression. For example, full-body expressions of fear communicate important information in an immediate, arousing, and contagious manner (de Gelder et al., 2004; Borgomaneri et al., 2015). Anger is commonly expressed with hands in fists, disgust with head tilted slightly forward, fear with hands raised to protect the body, sadness with shoulders slumped, surprise with arms raised, shame with downward head tilt (Izard, 1971; Keltner, 1995), while pride includes headed slightly tilted back and hands on hips (Tracy and Robins, 2007). Notably, the expression of emotions such as embarrassment, pride, and shame can only be recognized when body movements are combined with facial expressions. Body postures do, in fact, influence both the expression and the recognition of emotions (Shan et al., 2007; Dael et al., 2012). This issue becomes relevant when considering emotions such as guilt and love, which lack distinctive facial signals: they may display recognizable nonverbal expressions if body position is considered.

To sum up, emotional experience does not manifest itself in facial configurations alone, but rather in multimodal expressions involving head movements, gaze, and the body. The close connection and continuity of facial expressions with postural and gestural cues, however, has historically remained in the background (Lott et al., 2022). In recent decades, interest in the study of emotional body expressions has steadily increased (Lenzoni et al., 2020; Poyo Solanas et al., 2020; Watson and de Gelder, 2020), leading to the development of data sets on the emotional body with dynamic stimuli (e.g., Troje, 2002; Atkinson et al., 2004; Alaerts et al., 2011; de Gelder and Van den Stock, 2011). However, studies presenting facial and body expressions together (e.g., Rosenthal et al., 1979; Thoma et al., 2013) or investigating how body movements can express spontaneous and posed displays are still scarce. To obtain a more complete evaluation of emotional functionality, the synergistic actions of many different facial and body muscles, as well as gaze, physiological correlates and self-reports should be investigated with a triangulation approach. Triangulation is a strategy adopted by cartographers to map a new territory: three known points are defined and based on those, the unknown point is identified. In this case, the integration of reliable data from three different sources (i.e., physiological, psychological and behavioral) will allow answering questions such as: What are the neurophysiological processes that underlie the expression of emotion? Which physical features are globally encoded? What would a comprehensive atlas of human emotions’ expression include?

In a meta-analysis of physiological responses associated with a wide range of distinct emotions, several positive emotions (e.g., amusement, awe, contentment, desire, enthusiasm — all of which would be grouped under “happiness” by the classical basic six approach) were found to have subtly distinct patterns of peripheral physiological response linked to unique biological substrates (Kreibig, 2010). In this light, psychologists have uncovered that positive and negative valence information can increase pupil dilation (Bradley et al., 2008), making this measure a suitable proxy for understanding emotional load (Sirois and Brisson, 2014) in conjunction with high-frequency heart rate variability (HF-HRV), a biomarker of vagal-mediated parasympathetic activity able to detect states of distress (Dell’Acqua et al., 2020; da Estrela et al., 2021). We argue that only the combined recording of different and complementary techniques will provide a comprehensive emotional taxonomy.

## Methodological limitations

Past research investigating emotional displays has mainly focused on the facial muscle activation occurring during an emotional event using manual coding approaches, such as the Facial Action Coding System (FACS; Ekman and Friesen, 1978; Ekman et al., 2002). Classically, two FACS coders decompose an observed expression into specific Action Units (AUs; i.e., contraction or relaxation of distinct facial muscles) that produce the movement, and their outcomes are eventually compared. Although this is the most widely used method to categorize emotion expressions, its primary drawback is that it analyzes each facial movement independently from other movements. Being many facial muscles closely related, they cannot move independently (Hao et al., 2018). Moreover, FACS codes have a fixed range of applications: for instance, they do not incorporate AUs for emotions like pride, which has a complex expression that involves the body as well as the face. Another weakness of this system is that being a human coder requires an extensive training and it is very time-consuming. A trained FACS operator can take hours to code 1 min of video data depending on the complexity and density of facial expressions.

To solve this issue, researchers created automatized algorithms (Chu et al., 2017; Martinez, 2017; Park et al., 2020), which work very well in the laboratory, when images can be controlled. However, their accuracy drops substantially when they detect less constricted facial expressions (Benitez-Quiroz et al., 2017). Moreover, both manual FACS coding by expert raters (van der Schalk et al., 2011) and automatic detection by means of computational algorithms (Lucey et al., 2010; Valstar et al., 2017) have been applied to dynamic expression databases only on the apex. The estimation at multiple time steps is vital because, in real life, expressions vary in intensity over time.

In this respect, in the last decade, Machine Learning (ML) has been applied to both static and dynamic emotional stimuli to investigate the possibility to automatically discriminate emotions basing on facial expression configuration. ML is one of the most promising fields in the area of artificial intelligence (Mitchell, 1997): it is a discipline associated with computational statistics that aims to create new knowledge or predictions through algorithms that - based on real observations, categorizes items into different categories. In particular, ML algorithms are trained on a portion of data (training set). Then once trained, they are tested on the remaining data (test set). Besides being applied in emotional discrimination, ML algorithms have also been recently applied to discriminate genuine from posed emotional expressions. The first example regarded pain (Bartlett et al., 2014): the authors showed that, while human observers could not discriminate spontaneous from posed expression of pain better than chance (55% of accuracy), ML algorithms that automatically detected facial movements were able to achieve 85% of accuracy. Similarly, in another study (Monaro et al., 2022), authors recorded participants’ face while they recalled a real or posed emotional event. Again, the ability of ML algorithms to discriminate the true from the false story based on facial movements was much higher than the one of human beings (78 vs. 57%). These pioneering studies suggest that, when relying on facial cues only, artificial intelligence performs better than humans in discriminating genuine from posed emotions even if humans have more information to rely upon.

**Figure 1:**
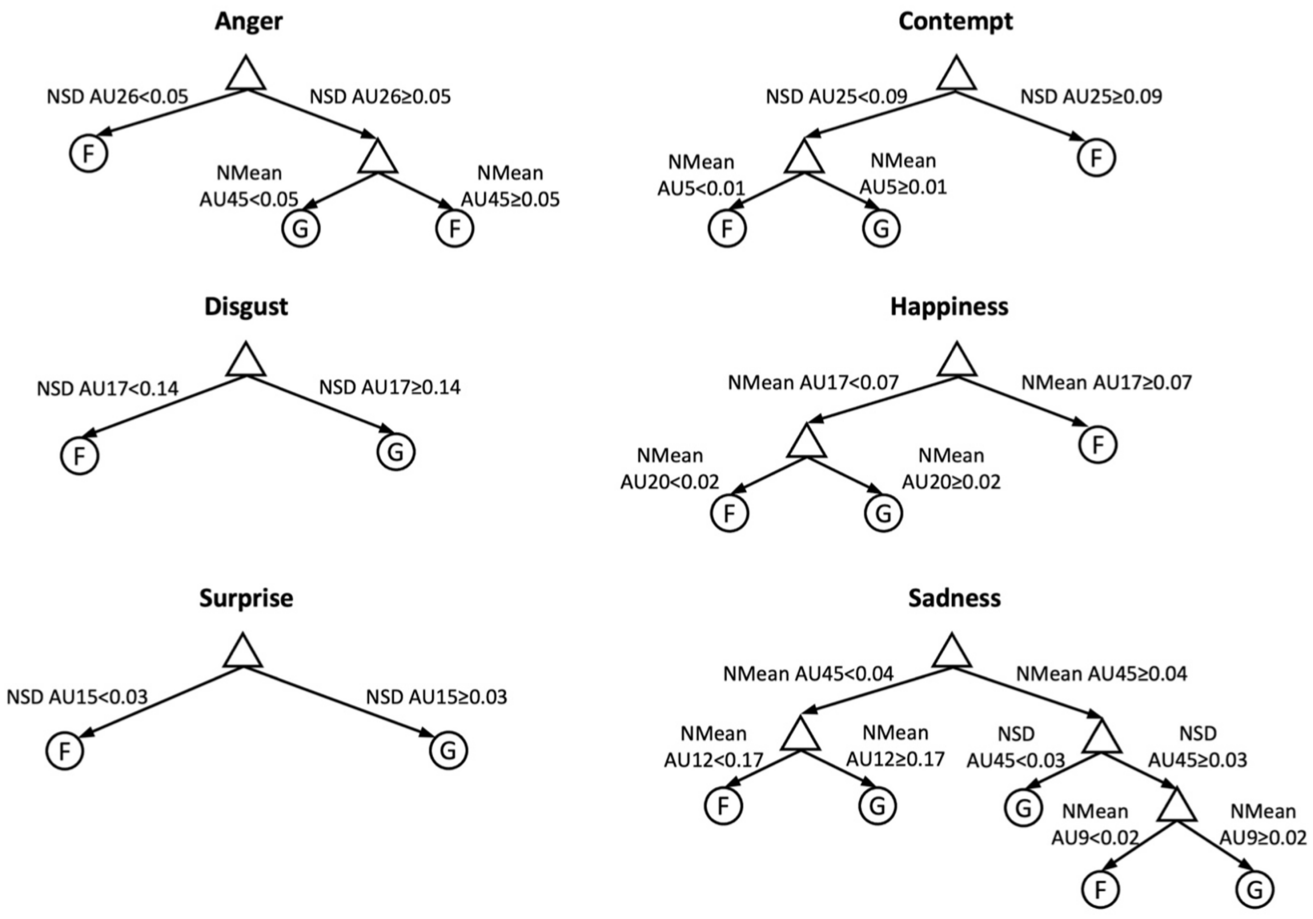
Best Tree models and normalized features (Nmean, NSD). Graphs report tree decision paths and feature thresholds to predict fake (F) and genuine (G) expressions. Image is modified from Cardaioli et al. (2022).

Despite ML algorithms are considered a powerful tool, their use is not free from criticisms. Nowadays, a widespread criticism to Machine Learning (ML) algorithms is that they provide un-interpretable results (i.e., a percentage of classification accuracy without explaining the classification rules; see Carvalho et al., 2019). Recently, a paper tried to overcome this shortcoming by using interpretable ML models able to detect and describe differences between genuine and non-genuine emotional expressions (Cardaioli et al., 2022). Interpretable ML models are algorithms that, besides providing the scientists with a classification accuracy, also identify facial movements that mostly contribute to the classification of genuine and posed emotions. In Figure 1, for instance, are reported the results of decision tree ML algorithms with a mean of 82% of accuracy in discriminating genuine and posed emotions. Moreover, decision tree, identifies, for each specific emotions, the AUs critical for the classification of genuine and posed, thus providing important insights for the neuroscientific understanding of emotions.

Although several studies have reported promising detection accuracy with intra-dataset testing scenarios, another drawback of ML application is that the performance can vary widely applying the same detection method to different databases (Jia et al., 2021). The weak consistency among the results may be due to the high inter-individual variability in the facial displays of emotions (Holberg et al., 2006; Sangineto et al., 2014; Durán et al., 2017). In general, the datasets used for training models do not adequately consider the real-world scenarios variability, an effect called “dataset bias effect” (Khosla et al., 2012). Although researchers have strong incentives to reduce the impact of individual differences as much as possible (e.g., to increase effect size and improve statistical power), the inter-variability among individuals should not be neglected in favor of a more generalist approach. Facial displays are not identical for different subjects, nor even for the same emotion (Sadeghi et al., 2013; Sangineto et al., 2014; Durán et al., 2017). A recent paper (Cardaioli et al., 2022) capitalized the PEDFE dataset described above (Miolla et al., 2022). The PEDFE dataset is unique to explore inter-individual differences in emotional expression, as, besides including genuine and posed dynamic emotional expression, it also includes many emotional stimuli for each “actor,” where the same emotion is expressed with different intensities or response to different stimuli. This allows to have a wide range of genuine expressions of the same emotion for each participant and to test the ability of ML models to discriminate genuine and posed emotions at the level of the single individual. An overall accuracy of 84.4% was achieved when applying ML models at the level of the single individual (i.e., for each subject, ML models can correctly discriminate genuine or posed emotions in the 84% of cases), as compared to the 67.0% when applying group-level algorithms. In general, these results suggest that it could be more reliable to detect unique deceptive cues for each subject instead of identifying a common rule to discriminate spontaneous and posed emotional facial expressions.

Recently, ML techniques have been used by Cowen et al. (2021) to address the debate about universal facial expressions by analyzing more than 6 million YouTube videos. The researchers used a powerful ML method involving deep neural networks (DNNs) to assess the extent to which specific facial configurations could be reliably observed in the videos across cultures. They found that people around the world make similar facial expressions in similar social contexts. Needless to say, the result is extremely interesting. On the other hand, there is no guarantee about the actual emotions felt by the people in those videos. A marriage context, for example, may lead one to believe that the emotions manifested are of joy. But this is all to be proven. In fact, the DNN learnt from human evaluators, who annotated the facial movements contained in each videoclip by choosing from a set of English words. The raters were, in effect, offering inferences about the emotional meaning of the facial movements (Barrett, 2021). Data science and algorithms can work very well on large numbers, but if the source lacks an accurate and reliable indication about the real emotion experienced by people, the whole analysis is tainted. In general, the great limitation of ML technology is that how you train the algorithm will determine the outcome, in a self-referential way. That is why we propose to train ML algorithms with large - already separated - datasets of authentic and posed expressions, scientifically controlled and rich in psychophysiological information (e.g., ECG, EDA, self-report). Google can offer emotion science huge sets of real, but uncontrolled expressions, totally devoid of psychophysiological correlates and distorted by the personal beliefs of the researcher who selected the specific stimuli for the experiment. The solution we propose is to make a major collective effort at the level of the scientific community to create large, rich, multifaceted ecological datasets to take full advantage of the enormous potential of ML. Once these datasets have been acquired in controlled environments, it will be possible to use them to train algorithms that can also function in natural environments, which are by definition less controlled. Indeed, the ultimate goal of this process will be to create increasingly complex algorithms capable of extracting key features even from data collected in natural scenarios. Including dynamic and interactive information instead of limiting the science of emotions to an “individual peak” would be important in modern emotion research, especially considering today’s technological capabilities. We believe that only accurate triangulation of physiological, behavioral, and self-report data can ensure accurate identification of the emotion experienced. And only this data can then be properly used by the ML for the generation of predictive algorithms.

**Figure 2:**
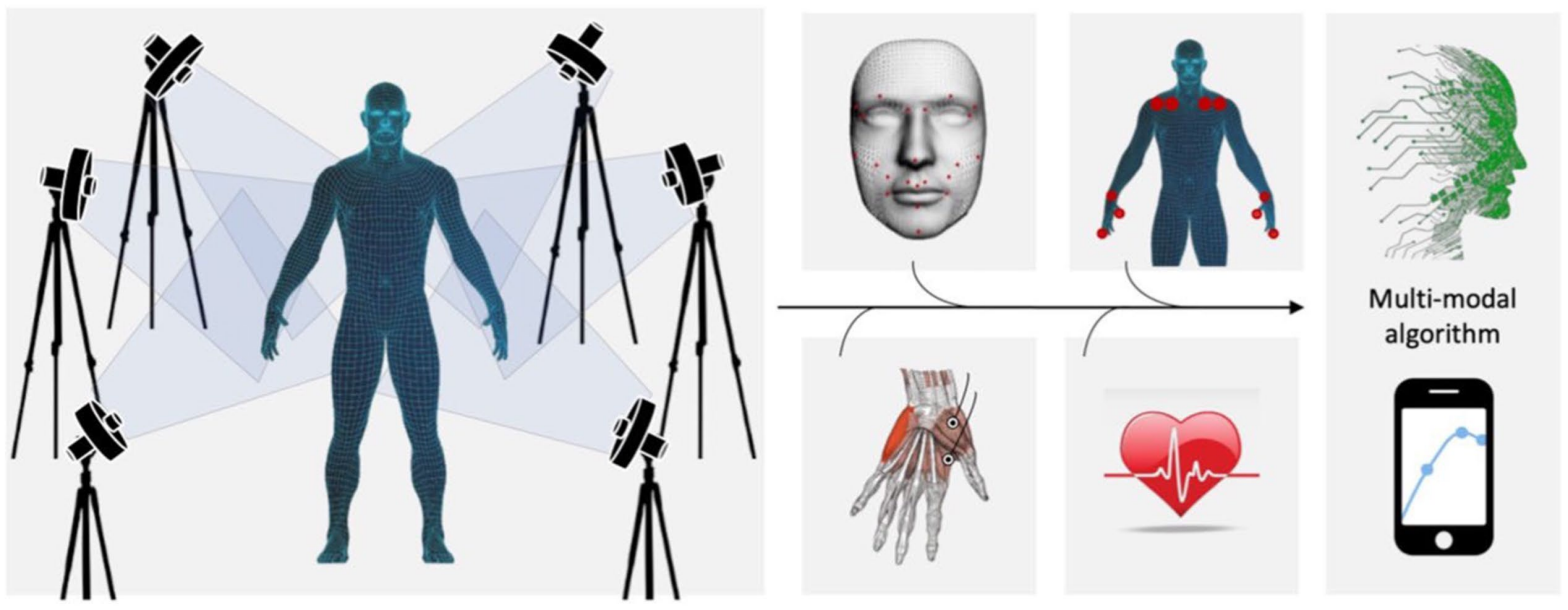
We propose an integrated methodology (i.e., kinematical 3-D analysis of movement, pupil dilation, gaze, EMG, heart rate variability, self-reports, and machine learning) to extract multi-modal detection algorithms able to discriminate the specific patterns of a wide range of blended emotion displays even in ecological contexts (e.g., with smartphone Apps).

To sum up, these results indicate that both manual and automated coding have temporal, spatial and reliability limitations. We suggest that the true move towards an objective analysis of emotional function will begin with the 3-D tracking of small configurations of points (landmarks) to define a unique set of universal and easily recognizable reference points for extracting the kinematics of face and body movements in a replicable manner. A simple model would allow to analyze separately the upper and lower face and to compare the left and right faces (see Figure 2). Moreover, considering the relative position of couples of points instead of single points would allow to neutralize possible head movements. This methodological step is nowadays crucial, since the variety of evaluation methods and evaluated movements that are present in the literature does not suggest a unique and easily applicable standardized method. An exhaustive quantitative analysis of facial and body motion kinematics shall then be integrated with EMG (Hess et al., 2017; Beringer et al., 2019), physiological indexes such as pupil dilation (Brod, 2021), gaze (D’Mello et al., 2012), heart rate variability (Dell’Acqua et al., 2020; da Estrela et al., 2021), and self-reports (Durán et al., 2017). Such a fully integrated database would allow to extract multi-modal detection algorithms able to discriminate the specific patterns of a wide range of blended emotion displays, and to assess the efficiency of emotional expressivity also in pathological conditions (e.g., ASD syndrome). Interestingly, these algorithms would be easily applicable in ecological contexts (e.g., with smartphone Apps; see Figure 2) for the assessment of inter-individual and cultural differences.

New databases of genuine displays will also allow to overcome a critical issue related to emotion discrimination tasks (Barrett et al., 2019). Although the forced-choice paradigm classically adopted in emotion recognition tasks might yield robust results, it lacks ecological validity since it forces the use of labels that might not otherwise be selected. An essential improvement in the actual literature would be obtained by combining free-response tasks with the kinematic analysis of emotional movements, which may allow to better understand and describe participants’ emotional reactions. In the free-response tasks, participants would be allowed to decode the type of observed emotion into continuous emotion ratings as expressions progress over time, while judging dimensions of valence and intensity. Accurately assessing kinematic parameters in these tasks would then provide an implicit measure of the association between observed emotions and participants’ reactions (i.e., motor contagion; Borgomaneri et al., 2020).

## Directions for future research

One reason for the relative lack of previous research attention to whole-body displays is the absence of a precise navigation system for coding emotions from movements. Targeting the 3-dimensional space to expand our understanding of how facial and body displays unfold over time is therefore of primary importance. Studying the dynamic aspects of emotional expressions will open a new stage in understanding how they are expressed and recognized. Furthermore, we maintain that the most viable solution to start better addressing the ecological validity aspect is defining an innovative approach to emotional testing. Novel and more integrated databases of genuine dynamic stimuli will meet new demands in research on human communication, machine recognition, and human-computer interaction. This approach can potentially lead us to the roots of emotional display, and to the individual mechanisms underlying their expression (i.e., emotional signatures). Kinematics, moreover, might allow to disentangle, from a very innovative perspective, the relative role of experience and culture in shaping emotional expression. By opening up the field to a richer description, new fields of neuroscientific inquiry will consequently emerge.

In applicative terms, providing a new database that can span a large range of spontaneous emotion expressions would pave the way for human-computer interaction research (Pantic and Bartlett, 2007). Increasing efforts are nowadays targeted towards developing robotic systems able to recognize and respond to emotional signals, which can be applied in fields such as security, medicine, education, and digital communication. A high-dimensional taxonomy will open a new stage in understanding how emotional expressions are recognized, allowing to develop new algorithms which would be an alternative to those commonly used to detect emotions. Companies and governments are spending billions of dollars in trying to improve the way emotions are detected. Tech giants strive to improve algorithms designed to detect a person’s emotions to assess the suitability of job candidates, detect lies, make adverts more alluring and diagnose disorders from dementia to depression. Estimates place the industry’s value for this research at tens of billions of dollars. However, we shall look at the full picture, as faces alone do not reveal much about emotions. In the future, cooperative efforts between psychology and computer science are indispensable (e.g., Valstar et al., 2015). For knowledge transfer and dialogue to increase, researchers from both sides will have to embrace unique and rich stimulus datasets.

Creating a high-dimensional and total-body taxonomy of emotion expression will also offer invaluable information to programs that seek to train children who live with autism (Wieckowski and White, 2017) and other conditions defined by difficulties in representing and reading one’s own and others’ emotions (e.g., Alexithymia). Such a taxonomy will allow investigations throughout life span – from childhood to old age – and will allow to identify functionally-relevant biomarkers that can early reveal disease onset. For instance, in stroke patients - where the hemispheric damage translates into asymmetries in contralateral facial expressions of emotion.

At present, there is still no standardized method to evaluate the accuracy and efficiency of full-body emotion expressivity, which could help in diagnosis, treatment planning, and post-treatment follow-up (Trotman et al., 2005). The absence of an accurate and universally accepted grading system for assessing the severity of emotional impairment makes comparisons of results invalid.

Among the quantitative instruments recently developed for the assessment of emotional movements, 3-D motion analyzers appear the most suitable for the collection of data in a great range of patients. They allow a complete and detailed assessment of motion in all parts of the face and body, and quantitative data can be compared between and within individuals (Coulson et al., 2002; Mishima et al., 2004; Nooreyazdan et al., 2004). A full understanding of emotional expression requires an appreciation of a wide degree of variability in display behavior, both within and across emotion categories. By introducing the concept of “individual emotional performance,” researchers will also provide a reference to compare long-term performance.

## The domain of the unpredictable

A few years ago, Chris Anderson - the editor of Wired - wrote an article titled “The end of theory: the data deluge makes the scientific method obsolete” (Anderson, 2005). Anderson argued the provocative thesis that with the advent of digital and the computational capabilities of supercomputers, theory is now useless. He also claimed that correlation prevails over causation, and science can progress even without coherent models or unique theories. In short, why should we waste time searching for causal relationships that explain what happens in the world when it is intellectually less demanding to entrust the machine (e.g., AI, ML) with the search for highly effective correlations? Indeed, because the characteristic feature of the digital is to record everything it comes into contact with, humanity is now awash in an unprecedented deluge of data: a huge archive of all human life forms.

We speculate that it is precisely this immense growth of data that requires theories capable of governing what would otherwise be chaos from a cognitive point of view. No one would be satisfied to explain the workings of the universe by resorting to simple correlations, because at that point there would be no difference between an astronomer and an astrologer. Mankind has made huge progress thanks to experimental science and unique theories.

The fact that of many correlations we cannot understand the cause does not eliminate the need for the theory, quite the contrary. The social world and its emotions, in particular, has long been considered the domain of the unpredictable. But now that human actions can be recorded in minute detail, human behavior can be understood and interpreted. Here is where collaboration between researchers, philosophers and engineers becomes essential.

## Conclusion

A growing body of evidence suggests that each emotional display is a complex, multi-component, motoric event. Human face is never static, but it continuously acts and reacts to internal and environmental stimuli, with the coordinated action of the facial muscles (Calvo et al., 2018). Most research on facial expression, however, has used static and posed expressions as stimuli, obtained from standardized databases (for a review, see Calvo and Nummenmaa, 2016). Yet, dynamic changes in the facial expression of emotions are a particularly valuable source of information: they indicate changes in the emotional state of other individuals. Our understanding of such dynamic information and the corresponding dynamic expression databases are so far very limited (for a review, see Krumhuber et al., 2017). Moreover, no study has yet combined EMG with 3-D motion analysis to provide a full spatio-temporal characterization of blended emotional expression at both the muscular and kinematic levels. We propose that richer and larger data sets - including facial blends of emotions, will provide an ideal test case for studying emotion expressions. The search for this emotional taxonomy, coupled with more powerful quantitative approaches, will in turn allow a better understanding of the rules governing the syntax of facial expressions.

This knowledge might eventually have large implications in the strongly debated issue concerning the role of nature and culture on the expression of emotions. Note that this debate arose exactly 150 years ago, when Darwin (1872) proposed facial expressions of emotion to be universal. We believe it is time to overhaul the science of emotion with better tools and more valid experimental designs.

## Author contributions

All authors contributed to conception of the review. LS and ES wrote the first draft of the manuscript. CS wrote sections of the manuscript. All authors contributed to the article and approved the submitted version.

## Funding

The present work was carried out within the scope of the research programme “Dipartimenti di Eccellenza” (art.1, commi 314-337 legge 232/2016), which was supported by a grant from MIUR to the Department of General Psychology, University of Padova.

## Conflict of interest

The authors declare that the research was conducted in the absence of any commercial or financial relationships that could be construed as a potential conflict of interest.

## Publisher’s note

All claims expressed in this article are solely those of the authors and do not necessarily represent those of their affiliated organizations, or those of the publisher, the editors and the reviewers. Any product that may be evaluated in this article, or claim that may be made by its manufacturer, is not guaranteed or endorsed by the publisher.
